# 90-90-90 cascade analysis on reported CLHIV infected by mother-to-child transmission in Guangxi, China: a modeling study

**DOI:** 10.1038/s41598-020-62281-8

**Published:** 2020-03-24

**Authors:** Gang Wang, Chunyan Lu, Shanfang Qin, Wudi Wei, Jingzhen Lai, Junjun Jiang, Bingyu Liang, Oulu Zhou, Jing Han, Yao Yang, Li Ye, Hao Liang, Chuanyi Ning

**Affiliations:** 1 Guangxi Collaborative Innovation Center for Biomedicine, Guangxi Medical University, Nanning, 530021 Guangxi China; 2 Guangxi Key Laboratory of AIDS Prevention and Treatment, Guangxi Medical University, Nanning, 530021 Guangxi China; 3 Life Science Institute, Guangxi Medical University, Nanning, 530021 Guangxi China; 4 Nursing College, Guangxi Medical University, Nanning, 530021 Guangxi China; 5 School of Public Health, Guangxi Medical University, Nanning, 530021 Guangxi China; 6 Guangxi Center for Disease Prevention and Control, Nanning, 530021 Guangxi China; 7 Longtan Hospital of Guangxi Zhuang Autonomous Region, Liuzhou, 545005 Guangxi China

**Keywords:** HIV infections, Health policy

## Abstract

The prevalence of HIV in Guangxi was very high, and there were many children living with HIV (CLHIV) because of larger baseline of pregnant women infected by HIV. It is necessary for children to explore the status of antiretroviral therapy (ART) on different initial CD4 counts in children with HIV infected by mother-to-child transmission (MTCT) in Guangxi and to evaluate the progress towards the 90-90-90 targets proposed by UNAIDS/WHO. Based on a retrospective observational cohort of children with HIV infected from the Guangxi Center for Disease Prevention and Control (CDC), the variables of all patients included viral loads, CD4 counts, laboratory results and WHO clinical staging of HIV/AIDS were collected. Several indicators were defined before analyzed: (1) diagnosis of MTCT: infants born to HIV-positive mothers who tested positive for HIV twice before 18 months; (2) ART initiation: the children who were enrolled in the treatment cohort and were still having HIV monitoring as of 6 months before date censored and (3) viral suppression: a recently viral load measurement that was less than 1000 copies per milliliter. The number of CLHIV in Guangxi was projected by using the estimates of the national HIV/AIDS prevalence from China CDC. An Autoregressive Integrated Moving Average (ARIMA) model and the Holt Exponential Smoothing (ES) model were used to predict the number of CLHIV, the diagnosed CLHIV, the diagnosed CLHIV receiving ART and the number of them achieving viral suppression, in 2019 and 2021, respectively. In this 14-year HIV/AIDS treatment cohort, 807 children who were HIV infected by MTCT were enrolled. The ARIMA and Holt ES models showed that by the end of 2019, 82.71% of all CLHIV in Guangxi knew their HIV status, 84.50% of those diagnosed had initiated ART, and 85.68% of those on ART had durable viral suppression. By the end of 2021, 93.51% of all CLHIV in Guangxi will know their HIV status, 84.28% of those diagnosed will have initiated antiretroviral therapy, and 85.83% of those on ART will have durable viral suppression. Therefore, in 2021, Guangxi fails to achieve the WHO/UNAIDS 90-90-90 targets for CLHIV, and there is still a wide time interval between the first HIV-positive diagnosis and ART initiation. National free antiretroviral treatment program (NFATP) requires strong enforcement to reduce the prevalence of later chronic diseases and complications.

## Introduction

The epidemic of HIV/AIDS has brought tremendous challenges to human life worldwide^1^. There were 37.9 million [32.7million–44.0million] people living with HIV (PLHIV) according to the UNAIDS in 2018; with regard to children, approximately 2.1 million children were living with HIV, but only 43% of them received antiretroviral therapy^2^. The prevalence of HIV/AIDS contributed to the increase of morbidity and mortality in children, from which about one-sixth of the deaths from HIV infections were in children younger than 15^3^. China was a developing country which showed a low prevalence of HIV/AIDS, but the number of new infections was high (an average of 84000 cases report a year from 2014 to 2018). From the regional distribution of HIV/AIDS in China, the prevalence showed a strong clustered distribution, while Guangxi was a hot spot with high incidence^4^, ranking the second in China^5^. According to the Center for Public Health Science Data, the incidence of HIV/AIDS in China was 3.97 (per 100000 populations) in 2016, while 12.48 (per 100000 populations) in Guangxi. Moreover, the prevalence of HIV/AIDS in pregnant women was 55.30 (per 100000 populations), and the timeliness and patient compliance of the mother-to-child block was poor^6^.

In 2014, the UNAIDS/WHO proposed that 90% of all PLHIV should know their HIV status, 90% of those diagnosed should receive ART, and 90% of those on ART should have durable viral suppression^7^. The 90-90-90 targets prioritize equity across populations, with special emphasis on children and adolescents^8^. Routinely assessing country-level and regional progress towards 90-90-90 targets is critical for HIV/AIDS intervention and strategic adjustment^9^. In 2016, the WHO updated the consolidated guidelines on the use of antiretroviral drugs for treating and preventing HIV infection, which proposed that all adults, adolescents and children with HIV/AIDS should receive ART regardless of CD4 counts and WHO clinical staging^10^. Despite this remarkable progress, there are still challenges to achieve 90-90-90 targets in HIV-positive children^11,12^, especially in resource-limited Guangxi, China. As an HIV-hit region in nearly 3 decades, the incidence of HIV/AIDS is still rising in Guangxi, which was cited as a major public health problem. Hence, although the rate of MTCT in Guangxi was consistent with other provinces, there were many children living with HIV (CLHIV) because of larger baseline of pregnant women infected by HIV. It is necessary to explore the status of initiating ART and evaluate the regional progress. Therefore, analyzing the CLHIV in Guangxi, we estimated the achievements of 90-90-90 targets in 2019 and 2011. The finding of this study may provide evidence in Guangxi when to achieve the 90-90-90 targets across the HIV care continuum.

## Methods

### Data sources

We mainly used a retrospective observational cohort from Guangxi CDC. The children case registry database and ART treatment database were included in this cohort. The case registry database contained basic information of diagnosed CLHIV, methods of diagnosis, WHO clinical staging, viral load, CD4 counts, therapeutic regimens and complications, etc. The ART treatment database included all the follow-up records of each child, such as viral load, CD4 counts, laboratory results and therapeutic regimens, etc. As of 807 cases were contained in case registry database and more than 21800 follow-up records were included in ART treatment database at the end of 2017.

### Inclusion criteria

The belief inclusion criteria were as follows: (1) the children were newly infected by HIV through MTCT; (2) the diagnosed CLHIV aged younger than 15 years; (3) laboratory indicators registered in Guangxi CDC were complete and (4) the children were diagnosed between 2004 and 2017.

### Measures

The number of CLHIV who know their HIV status in Guangxi was estimated based on the national estimates in 2005, 2007, 2009 and 2011, respectively. The number of diagnosed PLHIV per year were obtained from two public databases, the Center for Public Health Science Data (http://www.phsciencedata.cn/) and the Guangxi Zhuang Autonomous Region Health Committee (http://www.gxhfpc.gov.cn/xxgks/yqxx/yqyb/). Secondly, combined with the national HIV estimated from 2005 to 2011 and newly diagnosed cases per year in China and Guangxi, the number of PLHIV in 2013, 2015 and 2017 were projected. To verify accuracy of this process, we compared the estimated values calculated by this method with the national estimate in 2018^13^, the results were consistent. Subsequently, the proportion of mother-to-child transmission in different years were obtained through literature review^14,15^. The number of CLHIV in Guangxi was estimated as follows: CLHIV in Guangxi was the number of the national estimate multiplied by the number of patients in Guangxi divided the number of patients in China multiplied by the constituent ratio of MTCT in Guangxi (%). Finally, the proportion of CLHIV who knew their HIV status [diagnosed/infected (%)] was calculated.

The proportion of diagnosed CLHIV initiating ART [treated/diagnosed (%)] was estimated using the retrospective observational cohort extract form the children case registry database. A statistical weighting method was applied to those in-care children who were out of care previously but returned recently, so that all diagnosed children, regardless of their status, were included in the denominator^16^. Briefly when calculating the number of diagnosed children initiating ART in 2005, we calculated the time interval between the last care visit before 2005 and the first care visit in 2005. If the interval was <1 year, the child received a weight of 1, meaning that the child was in regular care and definitely included in 2005. If a child had a last visit exactly 2 years before first visit in 2005, the child received a weight of 2, which means that he or she represented not only a child initiating ART, but also another out-of-care child.

**Figure 1:**
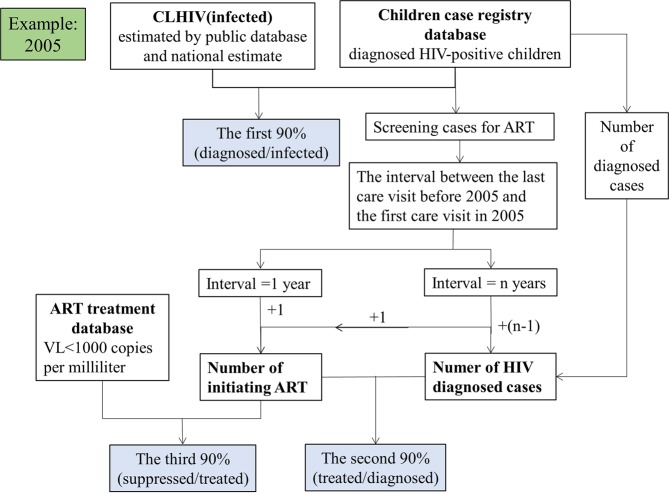
The pattern diagram of calculating 90-90-90. (2005 as an example).

The third 90% [suppressed/treated (%)] was estimated based on the diagnosed children who initiating ART, with the viral suppression status (fewer than 1000 copies per milliliter) obtained from the ART treatment database^17^. In other studies, viral suppression was defined as occurring when HIV was undetectable in the peripheral blood which less than 50 copies per milliliter^18,19^, it depended on the ability of the testing equipment to detect the virus. In China, we used the viral load fewer than 1000 copies per milliliter as the standard^20^. The detailed calculation process was shown in Fig. 1.

As for the assessment of 90-90-90 in 2019 and 2021, the ARIMA model and a Holt exponential smoothing (ES) method were used to predict the number of CLHIV, the diagnosed CLHIV, the diagnosed CLHIV receiving ART and the number of them achieving viral suppression in 2019 and 2021, respectively^21–24^. The ARIMA model is the most classic method for analyzing nonstationary time series. Establishing the ARIMA model consisted of four steps: (1) time-series data were obtained; (2) the data were drawn to observe whether the series was stationary; (3) the autocorrelation function (ACF) analysis and the partial autocorrelation function (PACF) analysis were employed to determine the parameter values including *p, q, P* and *Q*; and (4) the Akaike information criterion (*AIC*) and the Schwarz Bayesian information criterion (*SBC*) and ${R}^{2}$ were used to determine the optimal model^25^. The model with the lowest AIC and SBC values was considered the best model. If the AIC and SBC values of these plausible models were nearly equal, the model with the higher R^2^ value was selected. This method has been previously described elsewhere in detail^21^. The Holt ES model is simple and reliable to operate, especially for data that change continuously over time^23,24^. The establishment of the ES model consisted of three steps: (1) the initial values were determined; (2) the smoothing factor-alpha was selected; and (3) the predictive values were obtained using the optimal smoothing factor-alpha.

### Data analyses

Data were mostly identified and analyzed using JMP.14 Pro software. Briefly, screening diagnosed CLHIV from the children case registry database and ART treatment database according to the inclusion criteria. The ‘split’ function in software was used to divide the dataset into 7 subsets according to calendar year (2004–2005, 2004–2007, 2004–2009, 2004–2011, 2004–2015 and 2004–2017). Using ‘filter’ function to extract the interval between the first HIV-positive diagnosis and ART initiation per year. The medians (Interquartile range, IQR) were calculated to describe CD4 counts and WHO HIV clinical-stage, etc. Heat map and bar charts were plotted using GraphPadPrism7. The ARIMA and Holt Exponential Smoothing models were constructed by JMP.14 Pro, and line charts of predictive values were plotted by Origin 9.

### Ethical statement

The National Health and Family Planning Commission decided that the collection of data from cases of children living with HIV was part of a public health investigation, and thus the investigation was exempt from institutional review board assessment. The dataset was anonymised in the national reporting system except for individuals with special access, and it was anonymised for all before data analyses. The names and identifiers were removed when we obtained the dataset.

## Results

In this 14-year HIV retrospective observational cohort, 807 children who were infected with HIV-1 by MTCT were enrolled. The median age was 39 months (IQR: 22–65 months), and the median age of initiating ART was 53 months (IQR: 29–82 months). The CD4 counts in 291 (36.1%) children were fewer than 200 cells per microliter when newly HIV-positive diagnosed. 113 children (14.0%) had reached WHO HIV clinical stage 4 when diagnosed. The first line ART regimens included nevirapine, efavirenz (61.2%) or ritonavir-boosted lopinavir (37.5%), with only 1.3% of diagnosed CLHIV treated with single or two nucleoside reverse transcriptase inhibitors (zidovudine, stavudine or lamivudine) (Table 1).

**Table 1:** Baseline characteristics of the retrospective observational cohort.

Variables	n (%)
Sex	807
Male	422 (52.3)
Female	385 (47.7)
CD4 counts (cells per microliter)	803
<200	291 (36.1)
200–350	106 (13.1)
350–500	103 (12.8)
>500	303 (37.5)
Median (IQR)	358 (100, 717)
WHO HIV clinical stage	807
1	433 (53.6)
2	97 (12.0)
3	164 (20.3)
4	113 (14.0)
Median (IQR)	1 (1, 3)
Age at the time of diagnosis (month)	807
0–12	68 (8.4)
13–36	299 (37.1)
37–72	285 (35.3)
>72	155 (19.2)
Median (IQR)	39 (22, 65)
Age of initiating antiretroviral therapy (month)	807
0–12	62 (7.7)
13–36	206 (25.5)
37–72	287 (35.6)
>72	252 (31.2)
Median (IQR)	53 (29, 82)
Interval between diagnosis and initiation of antiviral treatment (month)	807
0–2	469 (58.1)
3–12	152 (18.8)
13–36	101 (12.5)
>36	85 (10.5)
Median (IQR)	1 (0, 11)
First line ART regimen	806
2 NRTIs + 1NNRTI	493 (61.2)
2 NRTIs + 1PI	302 (37.5)
Single or two drugs	11 (1.3)

**Figure 2:**
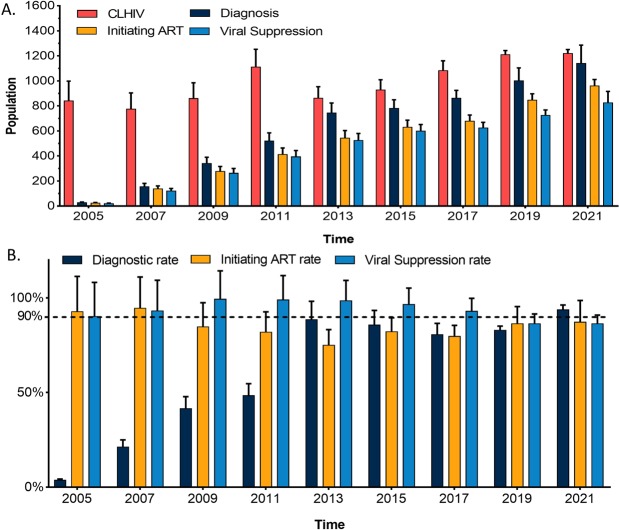
Progress of UNAIDS 90-90-90 targets in Guangxi. (**A**) The number of children diagnosed with HIV/AIDS from 2005 to 2017 respectively. In 2019 and 2021, the number of CLHIV were estimated using ARIMA model, and the number of diagnosis, on ART and viral suppression were estimated using Holt ES method; (**B**) the progress of the 90-90-90 targets in Guangxi per year. Black horizontal dotted line indicated Joint United Nations Programme on HIV/AIDS UNAIDS/WHO 90-90-90-targets. Red bars indicated the proportion of ‘the first 90%’ [diagnosed/infected]; green bars indicated the proportion of ‘the second 90%’ [treated/diagnosed]; and blue bars indicated the proportion of ‘the third 90%’ [suppressed/treated].

The information of the diagnosed CLHIV, the diagnosed CLHIV receiving ART and viral suppression were collected in 2005, 2007, 2009, 2011, 2013, 2015 and 2017 from the children case registry database and ART treatment database. The number of CLHIV in Guangxi was estimated based on the national estimates in 2005, 2007, 2009 and 2011, respectively. The results showed that 839 children (95% CI: 563–1115) in Guangxi were infected with HIV through MTCT in 2005, of which 28 children (95% CI: 19–37) were diagnosed. In 2007, 774 CLHIV (95% CI: 550–999) were estimated and the number of reported CLHIV was 155 (95% CI: 110–200). By 2009, there were 859 CLHIV (95% CI: 641–1076) and 341 (95% CI: 255–427) knew their HIV status. In 2011, the number of CLHIV was 1111 (95% CI: 865–1356) and the diagnosed children were 519 (95% CI: 404–634). After 2011, the number of CLHIV decreased slowly, while the number of HIV-positive diagnosed children increased (Fig. 2). The diagnosed CLHIV who initiating ART accounted for more than 90% between 2005 and 2007, while declined before 2013 and nearly increased to 90% thereafter. The rates of viral suppression remained above 90% from 2004 to 2017. The progress of WHO/UNAIDS 90-90-90 targets in Guangxi were showed in Fig. 2 and the estimated number of CLHIV in Guangxi was shown in Table 2.

**Table 2:** Estimated number of children living with HIV by MTCT in Guangxi.

Year	Population estimation		Reported PLHIV nationwide	Reported PLHIV in Guangxi	Estimated in Guangxi		Estimated CLHIV infected with MTCT in Guangxi	
	Value	95% CI			Value	95% CI	Value	95% CI
2005	485674	(325674, 645674)	179676	38795	104864	(70318, 139410)	839	(563, 1115)
2007	551503	(391503, 711503)	253429	44487	96812	(68725, 124898)	774	(550, 999)
2009	631639	(471639, 791639)	342086	58128	107330	(80142, 134517)	859	(641, 1076)
2011	724648	(564648, 884648)	444712	85193	138819	(108168, 169470)	1111	(865, 1356)
2013	857286	(697286, 1017286)	436817	54788	107525	(87457, 127593)	860	(700, 1021)
2015	1036598	(876598, 1196598)	577423	64455	115711	(97850, 133571)	926	(783, 1069)
2017	1258997	(1098997, 1418997)	758610	81386	135069	(117904, 152234)	1081	(943, 1218)

**Figure 3:**
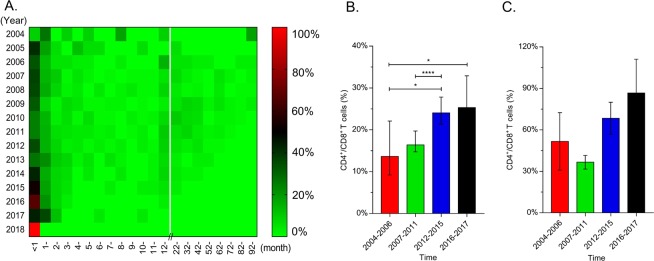
Other indicators to reflect the status of initiating ART. (**A**) Heat map of the interval between diagnosis and initiation of ART. Red, black and green indicate the constituent ratios at different intervals between diagnosis and initiation of ART per year; (**B,C**) CD4% (CD4+ T cells/CD8+ T cells) of children diagnosed with HIV. (**B**) Indicates the CD4% of children older than 1 year, which was grouped according to the periods of free ART policies changing; (**C**) indicates the CD4% of children younger than 1 year. All policies recommend that ART was prescribed free of charge for children younger than 1 year no longer considering the CD4 count or CD4%; therefore, the difference had no significant in CD4% under different policies (*P* > 0.05).

The intervals between the first HIV-positive diagnosis and ART initiation were shown in Fig. 3A. From 2004 to 2006, the median interval between diagnosis and ART was 2 months (IQR: 0–12 months) among 67 diagnosed CLHIV; and then the interval was 2 months (IQR: 0–19 months) from 2007 to 2011, with the adjustment of the free ART guidelines in China. Subsequently, the interval between diagnosis and ART was 1 month (IQR: 0–7 months) in 224 children from 2012 to 2015; and from 2016 to 2018, for the 70 children with HIV/AIDS, the median interval was fewer than 1 month (IQR: 0–1 month). In addition, the children were grouped by the adjustment time of free ART guidelines in order to explore CD4% (CD4/CD8). As shown in Fig. 3B, the median percentages were 13.62 (IQR: 7.09–26.88) from 2004 to 2006, 16.38 (IQR: 4.56–29.64) from 2007 to 2011, 24.08 (IQR: 11.47–43.43) from 2012 to 2015 and 25.30 (IQR: 6.05–47.38) from 2016 to 2018 among the children older than 1 year and the difference was statistically significant. In the children younger than 1 year, the lowest CD4% was 29.33 (IQR: 20.59–46.39) in 2007–2011 and the percentages increased thereafter.

**Figure 4:**
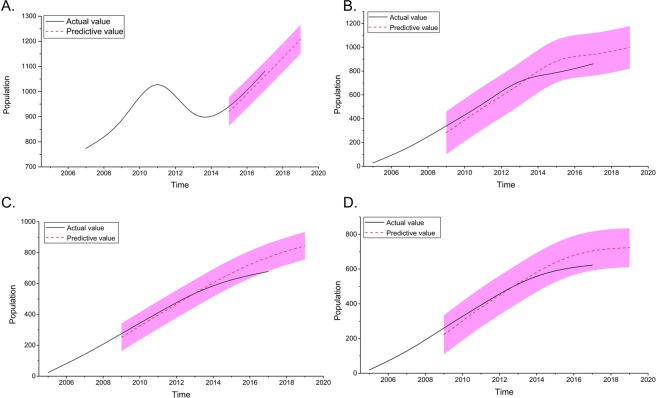
The forecast curves of the ARIMA and the Holt ES models as well as the actual HIV/AIDS series. (**A**) The actual values in 2005–2017 and the forecast curve of CLHIV in Guangxi using the ARIMA model; black line indicated the actual value, red dotted line indicated the predictive value, purple range indicated the 95% confidence interval (CI); (**B**) the actual values in 2005–2017 and the forecast curve of the reported CLHIV in Guangxi using the Holt ES model; (**C**) the actual values in 2005–2017 and the forecast curve of the children diagnosed with HIV of initiating ART in Guangxi using the Holt ES model; and (**D**) the actual values in 2005–2017 and the forecast curve of the diagnosed children of viral suppression in Guangxi using the Holt ES model.

**Figure 5:**
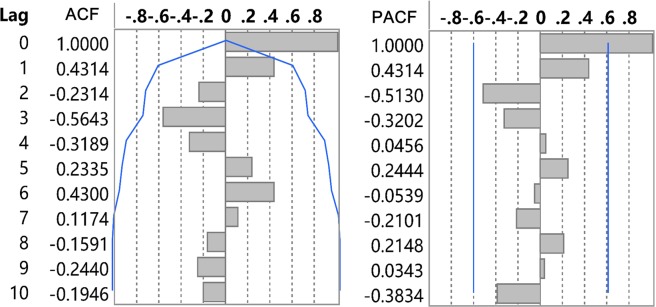
The ACF and PACF graphs of HIV series. ACF: the autocorrelation function graph; PACF: partial autocorrelation graph. The possible values of *q* and *Q* were 0, 1, 2 on the ACF graph and the possible values of *p* and *P* were 0, 1 and 2 on the PACF graph.

An autoregressive integrated moving average (ARIMA) model was developed to predict the population of CLHIV in Guangxi. As shown in Fig. 4, the number of CLHIV increased rapidly in 2011 and decreased thereafter. The ACF and PACF analyses were then performed to determine the parameter values (*p, q, P* and *Q*) of the ARIMA models (Fig. 5). Three appropriate models were identified to predict the population of CLHIV in 2019 and 2021. The model ARIMA (0, 0, 0)(0, 1, 0)_9_ had the lowest *AIC* and *SBC* values than the other two and it was selected as the optimal model (Table 3). The forecasting curve of the ARIMA and the Holt ES models and the actual values were shown in Fig. 4.

**Table 3:** The *AIC*, *SBC*, and *R*^2^ of the three appropriate ARIMA models.

Model	AIC	SBC	R^2^
ARIMA(0, 0, 0)(0, 1, 0)_9_	19.7575	18.4506	0.7237
ARIMA(0, 0, 0)(0, 1, 0)_8_	30.6497	29.7483	0.7947
ARIMA(0, 0, 0)(0, 1, 0)_7_	41.0112	40.3975	0.8816

*AIC*: Akaike information criterion; *SBC*: Schwarz Bayesian information criterion.

## Discussion

In Guangxi, this is the first study to describe the therapeutic status of ART in HIV-positive children through MTCT using a retrospective observational cohort. We found that by the end of 2017, 79.68% of all CLHIV in Guangxi knew their HIV status, 78.75% of those diagnosed had initiated ART, and 91.89% of those on ART have durable viral suppression. Additionally, we predicted the number of CLHIV, diagnosed CLHIV, the diagnosed CLHIV receiving ART and the number of them achieving viral suppression in 2019 and 2021, and the results showed that by the end of 2019, 82.71% of all CLHIV in Guangxi will know their HIV status, 84.50% of those diagnosed will have ART, and 85.68% of those who have initiated ART will receive durable viral suppression; by the end of 2021, 93.51% of all CLHIV in Guangxi will know their HIV status, 84.28% of those diagnosed will have initiated ART, and 85.83% of those on ART will receive durable viral suppression.

China initiated free ART through the National Free Antiretroviral Treatment Program (NFATP) in 2002. The guideline required that before 2005, the diagnosed CLHIV could take free ART when their CD4% (CD4^+^ T cells/CD8^+^ T cells) was fewer than 15%^26^. And the criteria of the CD4% was revised every 3 or 4 years (20% in 2008^27^ and 25% in 2012^28^). While since from 2016, HIV-positive children need to receive free ART as soon as possible regardless their CD4% or CD4 counts^10^. As regard to children younger than one-year-old, the free ART could be performed as soon as possible without their CD4 counts or WHO clinical stage since 2005. Therefore, we grouped diagnosed children according to the time points of guidelines adjustment and found that the increasing tendency of CD4% was coincident with the free ART guidelines in different periods (Fig. 3). The heatmap also showed that the intervals between diagnosis and ART initiation were shorter and shorter from 2005 to 2017, which facilitated the achievements of the second and third 90% targets.

The ARIMA model was developed to predict the number of CLHIV, and the Holt ES method was performed to forecast the number of diagnosed children initiating ART and viral suppression in 2019 and 2021. However, the reason why the diagnosed cases increased rapidly in 2011 is unclear. we think the reasons were as follows: (1) more HIV-positive people were treated with antiretroviral drugs, and thus reducing the mortality of HIV/AIDS (http://www.unaids.org/en/resources/campaigns/ together we will end aids/); and (2) the proportion of CLHIV that knew their HIV status was estimated based on the national estimates, so that the CLHIV in Guangxi was consistent with national tendency. Additionally, the ARIMA model obtained a predictive value by using a weighted average of the value from the past periods, so that the predicted results (Fig. 4) were characterized by periodicity rather than a consistently increase. As regard to the rate of viral suppression, which was lower (fewer than 90%) in 2019 compared with 2005–2017, the reasons might be that the free ART was taken in all HIV-positive children regardless their CD4 count or CD4% according to national free ART guideline in 2016, which led to the number of diagnosed CLHIV initiating ART increased rapidly.

Initiating ART for diagnosed CLHIV is very important for preventing and controlling in all age groups, because once children were diagnosed with HIV/AIDS, they will require lifelong treatment. Therefore, focusing on the HIV-positive children is a special focus on the entire population. Worldwide, there were still 220,000 new pediatric infections in 2014 although the rate of MTCT declined 41% reported in the past five years. About 55% [50–60%] of adults living with HIV knew their status, but only 32% of CLHIV were diagnosed^29^ and 37% of PLHIV received ART, while the proportion of diagnosed CLHIV on ART was 24%^7^. In other study, only half of HIV-exposed children received an early infant diagnostic (EID) test before two months of age in 22 Global Plan priority countries, although the WHO encouraged the scale-up of EID services^3^. And more than half (52.5%) of the CLHIV were dead younger than two years old without ART^30^. Therefore, initiating NFATP timely in infants and children is a life-saving preventive measure that down-regulates the incidence of subsequent chronic diseases and complications. As regard to the situation of less HIV detection rate in children, health-related departments should respond to national guidelines timely and improve screening process to identify PLHIV in the high-risk groups. Meanwhile, hospitals and healthcare institutions should prevent MTCT in HIV-infected pregnant women to cut the ‘denominator’ of the first 90-90-90 target [diagnosed/infected (%)]^31^. In addition, to increase the coverage of MTCT prevention, skills training and consulting services to pregnant women should be performed in order to raise compliance of subsequent intervention of MTCT. Community legal aid centers or legal services for cases of discrimination and human rights violations need to be reinforced, which are conducive to establish a mutual trust relationship among those who are discriminated against^32^.

In our study, several limitations also existed. Firstly, limited information was available and other information such as monitoring data, demographic data, the characteristics of high-risk and low-risk populations in Guangxi were not obtained^33^. It was difficult to estimate the number of CLHIV using some specialised models such as Spectrum, Workbook or Estimation and Projection Package (EPP)^34–38^. Moreover, the third 90% [suppressed/treated (%)] might be lower than the actual, because the latest guideline was revised in 2016, which requires the diagnosed CLHIV initiating ART as soon as possible, regardless of their CD4 counts. However, the effects of viral suppression did not emerge immediately, which might weaken the predictive effect.

## Conclusion

In 2021, Guangxi fails to achieve the WHO/UNAIDS 90-90-90 targets for CLHIV; and there is still a wide time interval between the first HIV-positive diagnosis and ART initiation. National free antiretroviral treatment program (NFATP) requires strong enforcement to reduce the prevalence of later chronic diseases and complications.

## Data Availability

The data is available.
